# Slowly progressive invasive rhino‐orbito‐cerebral aspergillosis: case report and literature review

**DOI:** 10.1002/ccr3.798

**Published:** 2017-01-26

**Authors:** Giselle de Martin Truzzi, Henrique Furlan Pauna, Igor Moreira Hazboun, Igor Benedick Coimbra, Emerson Taro Inoue Sakuma, Icléia Siqueira Barreto, Carlos Takahiro Chone, Eulalia Sakano

**Affiliations:** ^1^ Department of Otorhinolaryngology, Head and Neck SurgeryUniversity of Campinas (UNICAMP)CampinasSão PauloBrazil; ^2^ Department of Public HealthUniversity of Campinas (UNICAMP)CampinasSão PauloBrazil; ^3^ Department of RadiologyUniversity of Campinas (UNICAMP)CampinasSão PauloBrazil; ^4^ Pathological Anatomy DepartmentUniversity of Campinas (UNICAMP)CampinasSão PauloBrazil; ^5^ Head and Neck Surgery UnitDepartment of Otorhinolaryngology, Head and Neck SurgeryUniversity of Campinas (UNICAMP)CampinasSão PauloBrazil; ^6^ Rhinology UnitDepartment of Otorhinolaryngology, Head and Neck SurgeryUniversity of Campinas (UNICAMP)CampinasSão PauloBrazil

**Keywords:** Aspergillosis, fungal rhinosinusitis, nasal mass

## Abstract

This is a report of a patient with aspergillosis infection, which was thought to be a tumoral lesion during its investigation. This is not a common disease in Western countries, and this report should increase our awareness for differential diagnosis of nasal masses. Early diagnosis is desired in order to increase the survival rates.

## Introduction

Among the existing forms of rhinosinusitis, fungal invasive type is rare. However, its clinical relevance is due to a high mortality rate – ranging from 50% to 90% 1, 2 – and up to nearly 100% if intracranial mycotic dissemination is observed 3.

The *acute invasive form* is most found among immunocompromised patients because of their reduced capability to mount an effective response against fungal infections (marked neutropenia). It is characterized by an angioinvasive behavior, leading to necrosis of the affected tissues, and rapid evolution – possibly affecting the orbit, palate, and central nervous system (CNS), as the condition develops. The *chronic invasive form* tends to affect patients with subtler immune system abnormalities, as observed in uncontrolled diabetes mellitus (especially with diabetic ketoacidosis), prolonged corticoid therapy, and thyroid disorders 4. Its presentation is an indolent, prolonged course disease, with a better response to the treatment. The *granulomatous invasive form*, whose causal agent is usually the *Aspergillus flavus*, is mostly reported in Sudan, India, Saudi Arabia, and Pakistan. The course of the disease is quite similar to the *chronic invasive form*, but it is characterized by noncaseating granulomas, possibly with giant cells and signs of vasculitis, with sparse hyphae, histopathologically 5, 6.

## Case Presentation

A 65‐year‐old woman was referred to the Department of Otorhinolaryngology presenting a 5‐month history of a slowly progressive, oligosymptomatic orbital mass, causing left exophthalmia, left hemifacial pain, and ipsilateral visual impairment, which eventually evolved to total vision loss. The patient had an 8‐year history of uncontrolled type 2 diabetes mellitus and a stage IIB adenocarcinoma of the cervix which had been successfully treated with radiotherapy simultaneously with chemotherapy (cisplatin 40 mg/m^2^, weekly), 3 years before. Other comorbidities – adequately controlled with medication – included systemic hypertension, dyslipidemia, and hypothyroidism.

Physical examination presented a patient with left exophthalmia, vision impairment, and complete restriction of ocular motility. Anterior rhinoscopy and examination of the oral cavity were normal, as well of the neck with no alterations or masses. Nasofibrolaryngoscopy allowed perceiving a discrete medial bulging of the lateral wall of the left nasal fossa, and a nasal septal deviation to the left.

**Figure 1:**
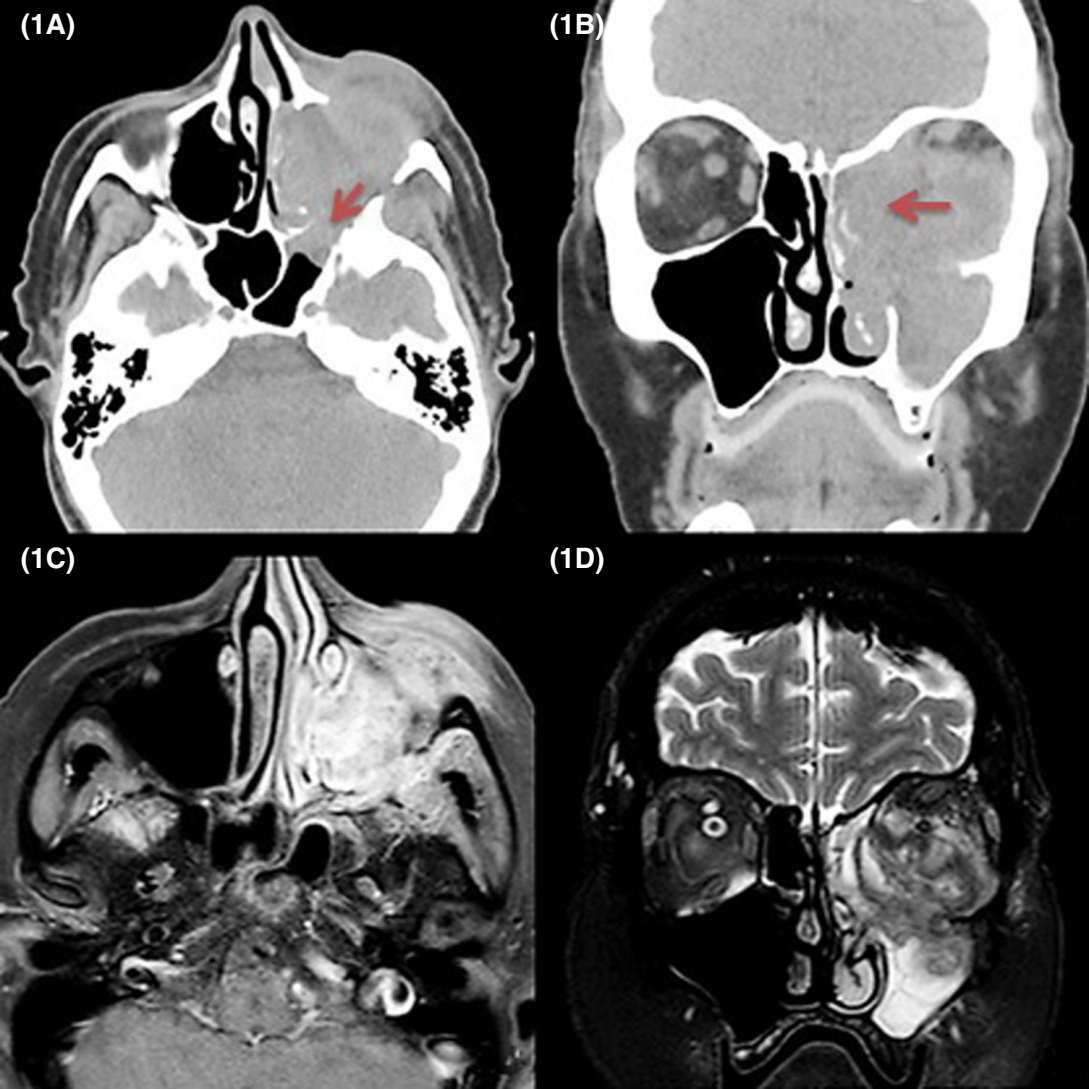
(A and B) Contrast‐enhanced, high‐resolution CT in axial and coronal plane images, respectively, showing a lesion with soft‐tissue density causing bone discontinuity of the maxilla, septal, sphenoid, ethmoidal, and pterygopalatine fossa (arrows). (C) T1‐weighted postcontrast MRI axial plane image showing a lesion with heterogeneous enhance affecting the left maxillary sinus, extending to the face. (D) T2‐weighted coronal plane image showing a heterogeneous mass in the region of the maxillary sinus, extending to the orbital region.

Computed tomography (CT) and magnetic resonance imaging (MRI) of the orbital area were obtained. The CT showed a lesion in the maxillary sinus with extension to the orbital region, ethmoidal and sphenoid sinuses (Fig. 1A and B). The MRI (Fig. 1C and D) showed heterogenic expansive mass in the topography of the maxillary sinus, nasal cavity, ethmoidal sinuses, and intraorbital region on the left, with signs of bone discontinuity in the left orbit's lateral and inferior walls. There was a deep extension through the inferior orbital fissure and signs of invasion of the cavernous sinus on the left. There were areas with restriction to diffusion semi‐circumferentially involving the ophthalmic nerve, and anterior deviation of the extrinsic ocular muscles and its blood vessels. Those findings were considered to be highly suggestive of malignant neoplasm.

**Figure 2:**
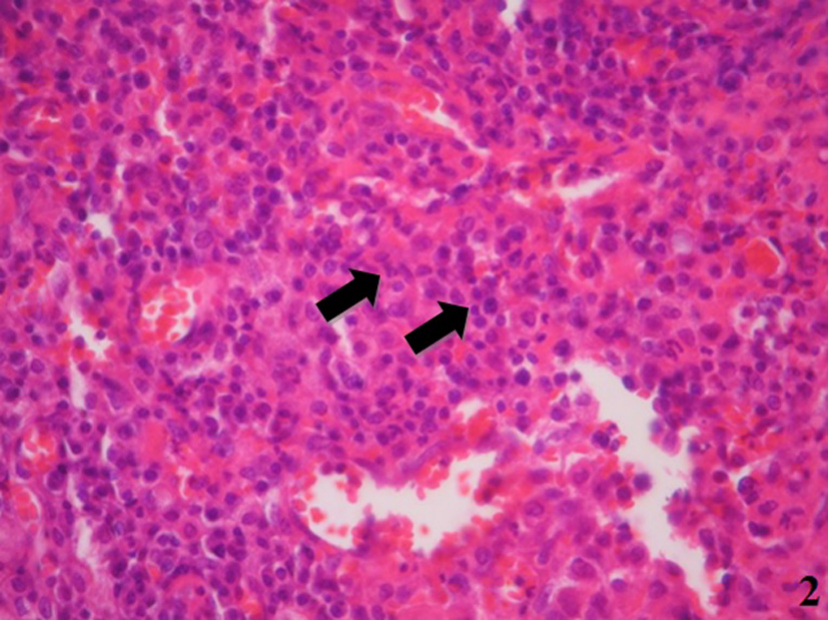
Image reveals nonspecific acute and chronic inflammation, without evidence of neoplasia or granulomas in the sample (H&E stain, 60X magnification).

During admission, blood samples and cerebrospinal fluid were collected to investigate infections, inflammation, neoplasia, or any other related disease (Tables 1 and 2). Also, the patient underwent to three consecutive biopsies. The first – right after the first presentation to the service – approaches the left maxillary sinus region through an endonasal endoscopic procedure. The analysis of the sample was released in four weeks, showing an intense lymphoplasmacytic proliferation that suggested a chronic, nonspecific inflammatory process in contrast to the probable diagnosis of a low*‐*grade lymphoproliferative lesion in the frozen section procedure. The paraffin fixation processing revealed nonspecific chronic fibrosing inflammation, without evidence of neoplasia or granulomas in the sample. No fungi were observed in the sample (Fig. 2).

**Table 1:** Laboratory values at admission and during disease course

	August	October	November
Capsular antigen *Cryptococcus neoformans*		Negative (latex method on blood and liquor)	
HbA1c (RV: 4–6)	8.8%		7.3%
CRP (RV < 0.3)		15.5	16.6
ESR (RV < 14)		120	
FTA‐ABS			NR
Hepatitis C			NR
HIV 1–2			NR
HBsAg			NR
Anti‐HBc			+269.65 mUI/mL

Anti‐HBc, total hepatitis B core antibody; CRP, C‐reactive protein; ESR, erythrocyte sedimentation rate; FTA‐ABS, fluorescent treponemal antibody absorption; HbA1c, glycated hemoglobin; HBsAg, hepatitis B surface antigen; HIV 1–2, human immunodeficiency virus antigen 1 and 2; NR, nonreacting; RV, reference value.

**Table 2:** Results from cerebrospinal fluid analysis

IgG (RV < 3.4)	11.3
Neoplastic cells	Negative
Fungi culture	Negative
Mycobacteria	Negative
Gram	Negative
FTA‐ABS	Negative
ADA (RV < 9)	1.8
Proteins (RV < 42)	58
Glucose	66
WBC (RV < 3)	5

ADA, adenosine deaminase; FTA‐ABS, fluorescent treponemal antibody absorption; RV, reference value; WBC, white blood cells.

**Figure 3:**
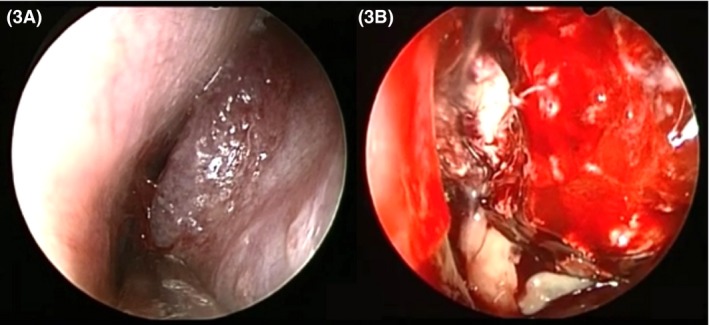
(A) Endoscopic aspect of the left nasal fossa, presenting no secretion. (B) Following orbital decompression, a large volume of secretion and necrotic tissue drained.

The second procedure, scheduled after obtaining the results from the first biopsy, involved endoscopic endonasal orbital decompression of the affected side. At that time, about 1 month after the patient's first contact with the institution, a granulomatous lesion was observed in the nasal fossa. During decompression, tissue of necrotic appearance was found, with drainage of a large volume of secretion (Fig. 3).

**Figure 4:**
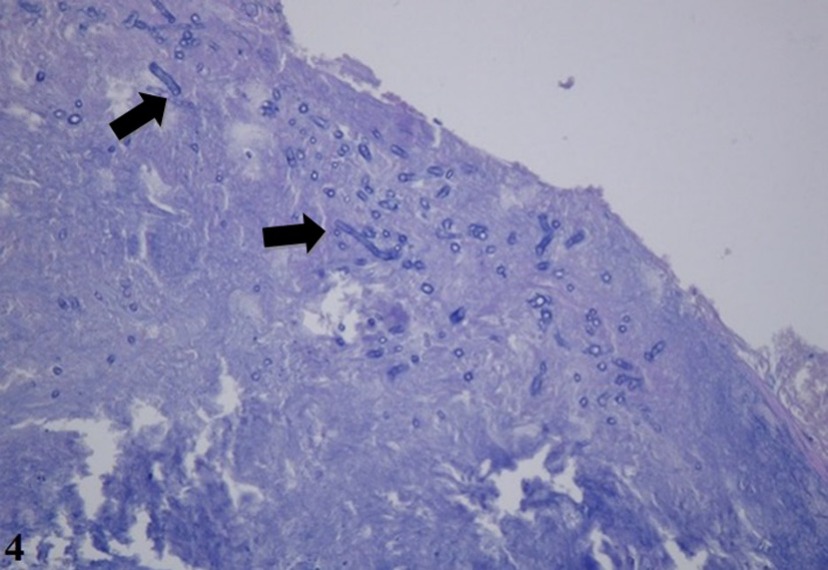
Aspergillosis detected under microscopic examination (arrows), without vascular invasion indicating fungal invasion. No neoplasia was found at the sample (PAS stain, 40X magnification).

Microscopic examination of the harvested material turned out suggestive of fungal infection (namely aspergilloma), with no signs of vascular invasion (Fig. 4).

**Figure 5:**
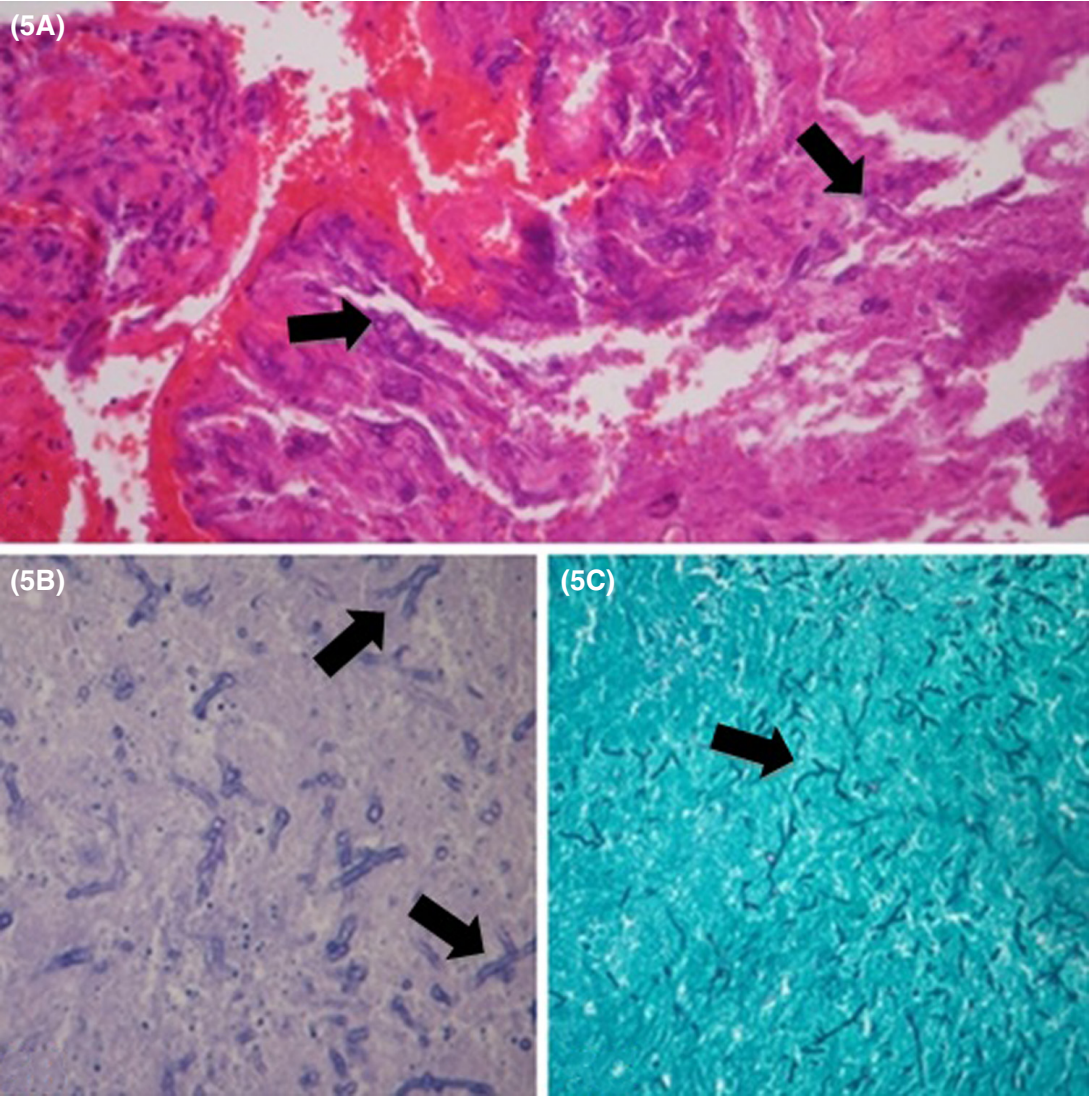
(A) Presence of numerous broad, septate hyphae of Aspergillus sp. (arrows) with characteristic branching at 45° angles (H&E stain‐40X magnification). (B) Aspergillus sp. hyphae (arrows) branching at 45° angles, highlighted by PAS stain (40X magnification). (C) At the image (10x magnification), multiple Aspergillus sp. hyphae (arrow) are visible, highlighted by Grocott stain, also with hyphae branching at 45°.

Meanwhile, approximately twenty days after the surgical decompression, the patient developed a contralateral amaurosis. A new MRI scan revealed an expansion of the lesion, which had extended into the anterior and middle cranial fossae on the left and into the cavernous sinus. Liposomal amphotericin B (IV, 5 mg/kg/per day) was initiated and kept for 20 days, but because there was no clinical improvement, a third biopsy (through a Lynch incision on the left) was then conducted, allowing the collection of large samples showing an active chronic necrotic inflammatory process, with dense accumulation of hyphae. The volume of the obtained sample was enough to definitively exclude neoplasm (Fig. 5). Cultured material identified *Aspergillus fumigatus*. Voriconazole (IV, 6 mg/kg/dose, every 12 h) was then initiated for treatment, under orientation of the hospital's Infectious Diseases Department. This triazole is also part of the therapeutic arsenal against aspergillosis, and the shift from amphotericin B was performed due to an overall deterioration of the patients clinical status and a progressive growth of the lesion with the latter antifungal drug.

During the entire hospitalization, the patient tended to sustain high serum glucose levels, despite of intensive monitoring and optimized insulin doses. Pathologies that could potentially cause glycemic abnormalities were investigated, but no etiological factor was found, except for the inflammatory process. The kidney function and liver transaminases were normal during the entire hospitalization, but hyponatremia without clinical repercussion was perceived during the investigation and readily corrected. No other radiological abnormalities were found, neither indication of other fungal infections elsewhere. The patient developed an acute ischemic stroke in the left carotid territory a few weeks after the initiation of voriconazole, presenting with a reduction in the level of consciousness and right hemiplegia. The patient remained under continuous intensive monitoring, having required mechanical ventilation and the use of vasoactive drugs. A encephalic CT scan showed a hypodensity in the territory of the left carotid artery, with a midline shift that did not require surgical decompression. The stroke eventually had a fatal outcome a few days later.

## Discussion

*Aspergillus*,* Bipolaris*,* Curvularia*,* Alternaria,* and *Rhizomucor,* among others, are agents possibly found in fungal rhinosinusitis 4, 5, 6. There are several reports of fungal rhinosinusitis and lesions associated with *A. flavus* in Iran and in the rural population in India 7, 8, 9. It is commonly seen in warm and dry climatic conditions, and it is particularly common in young men from rural background 10. The *Aspergillus* genre is becoming an increasingly recognized pathogen in the nasal tract 11. The *A. flavus* is most commonly associated with CNS infection in both chronic invasive and granulomatous rhinosinusitis, and frequently associated to vascular invasion, whereas the *A. fumigatus* is commonly involved with allergic fungal rhinosinusitis and aspergilloma 12.

Unlike *acute invasive forms*, the *chronic invasive form* tends to present as an indolent and oligosymptomatic infection, and symptoms may be present after weeks of progression 6. Initially, patients may report nonspecific symptoms – similar to chronic rhinosinusitis – such as headaches, nasal discharge, and facial pain. 7, 9. The most frequent complaints, as the disease progresses, are visual impairment and orbital apex syndrome 6. Complications related to the orbital extension include preseptal cellulitis, orbital cellulitis, subperiosteal abscess, and orbital abscess. The lesion grows contiguously, possibly invading the CNS – which increases the mortality rate. Another commonly described event that associates with a worse prognosis of the patients is the cavernous sinus thrombosis. Meningitis, encephalitis, and epidural, subdural and cerebral abscesses, as well as vascular emboli (or even an arterial occlusion) and infarctions are some other possible intracranial complications 13, 14. The negative outcome of the patient may have been caused by the aforementioned intracranial vascular complication, leading to a severe ischemic insult. The extremely oligosymptomatic development (which spammed over 10 months) observed in this report was atypical, which retarded the diagnosis.

Imaging examination shows a soft‐tissue mass; bone destruction and extension to the orbit, pterygopalatine fossa, or intracranial regions are possible findings. CT shows a hyperattenuating soft‐tissue mass with calcifications in the nasal sinuses. On MRI, the lesion may have iso‐ or hypointense T1 signal and is hypointense on T2‐weighted images. In some cases, it may be impossible to distinguish this mass from a nasal sinus neoplasm, as both may present bony wall destruction and extend beyond the affected nasal sinus 15, 16, 17. Other differential diagnosis includes carcinoma, sarcoma, lymphoma, juvenile angiofibroma, inverted papilloma, meningioma, neurofibroma, melanoma, and olfactory neuroblastoma (esthesioneuroblastoma) 18. The mentioned patient had imaging which emulated a neoplastic disease – the first diagnosis to be considered. The misleading image studies, combined to biopsies that were negative for typical findings of invasive fungal rhinosinusitis, lead to a retarded diagnosis – and hence treatment – of this patient.

The conclusive diagnosis is possible by histopathological examination with hematoxylin and eosin, periodic acid–Schiff (PAS), Grocot, and methenamine silver stains. The histopathology of the chronic invasive fungal rhinosinusitis is characterized by a dense accumulation of hyphae, infiltration of surrounding tissue 19, and vascular invasions (not found in noninvasive forms). Occasionally, there is also lymphocytic infiltration, presence of giant cells, granulomas, and necrotic tissue. A lesion may begin as a mycetoma and develop into the invasive form, especially in immunocompromised individuals 7, 9, 19, 20, 21. Some cases of allergic fungal rhinosinusitis may present tissue invasion associated with granulomatosis. In those cases, the most common associated pathogen is the *A. flavus* 12. In the presented case, the lack of vascular invasion was a misleading factor, retarding the correct diagnosis. Serum galactomannan was not used because it was not available in our service by the time of this case admission. It is also known the high rates of false positive and that is most indicated in cases of hematological malignancies 22. Characteristically, large, nonchambered hyphae with right‐angle branching are suggestive of *Mucor*, whilst *Aspergillus* shows smaller, chambered hyphae that branch at 45° angles 23, as presented by the patient in this report 24.

The optimal treatment consists of broad surgical debridement of involved tissues – provided it is clinically and technically possible – along with prolonged use of systemic antifungal medication (e.g., intravenous liposomal amphotericin B as a first choice, or voriconazole) and strict control of underlying medical disorders. Patients with neurological invasion have high mortality rates, requiring an aggressive treatment every time it is possible, similarly to the acute fungal invasive form. 20, 21. Patients should keep regular follow‐ups after the treatment, as the pathology is frequently recurrent.

## Conclusion

Chronic invasive fungal rhinosinusitis is a condition with complications of great potential morbidity and mortality. The clinical presentation may be poorly defined, and imaging characteristics are sometimes indistinguishable from nasal sinus neoplasms. The biopsy is the gold standard method to define the diagnosis, and early treatment allows a better prognosis.

## Authorship

GMT: main Author, was responsible for writing and reviewing the manuscript, literature review, and provided direct care to the patient. HFP: provided direct care to the patient, was responsible for writing the manuscript, and was responsible for the Discussion section. IMH: provided direct care to the patient and was responsible for writing the manuscript and table edition. IBC: involved in literature review, translator and corrector of the manuscript, and overall revision of the manuscript. ISB: was responsible for the anatomopathological examination and responsible for the pathology section of the article. ETIS: is radiologist, was responsible for the imaging procedures and case discussion, and was responsible for the radiological discussion. CTC: was responsible for the surgical care of the patient. ES: was responsible for conducting the patient's diagnostic and therapeutic approaches.

## Conflicts of Interest

The authors declare that there is no conflict of interest regarding the publication of this paper.
